# Quantifying Health State Utilities for Permanent Dentition: A Cross-Sectional Study

**DOI:** 10.1155/2022/1735011

**Published:** 2022-11-24

**Authors:** Yazeed Alharthi, Osama Alasmari, Hamad Almuaiqly, Saif Alhumaidi, Amjad Alemam, Wahdan Elkwatehy, Omair M. Bukhari

**Affiliations:** Umm Alqura University, Faculty of Dentistry, 2373 Al Awali, Makkah 24381, Saudi Arabia

## Abstract

**Introduction:**

Health utility represents individual preference strengths regarding health-related outcomes as a numerical value, with higher utility values of a health state achieved by a service or treatment strategy suggesting that it is more worthwhile to implement and allocate resources to this service. This study aimed to find and compare the utilities of permanent teeth-related health state outcomes.

**Materials and Methods:**

Two groups, one of the dentists (50) and another of dental patients (50), responded to a standard gamble questionnaire to determine the utilities of four hypothetical alternatives of dental health state as follows: (1) carious posterior tooth with pain, (2) carious posterior tooth without pain, (3) filled posterior tooth with a temporary restoration, and (4) filled posterior tooth with a permanent restoration. Values were calculated and compared between the two groups using the unpaired Student's *t*-test, and another comparison between gender groups was performed using a one-way analysis of variance.

**Results:**

There were significant differences between dentists and patients regarding health states 2, 3, and 4 (*p* = 0.011, 0.026, and 0.008, respectively). However, there were no significant differences between men and women regarding all health statuses. Nonetheless, there were significant differences between male dentists and male patients for health statuses 1 and 3 (*p* = 0.047 and *p* = 0.036), respectively.

**Conclusion:**

The oral health-related quality of life and its relation to economic dentistry is essential aspects of our modern practice. In the present study, there was a statistically significant difference in the utility value reported by dentists and patients. However, more research is needed in this area.

## 1. Introduction

During the localisation of healthcare services and expenses, the main impediment is the lack of outcome value. There is no accurate way to compare operative and endodontic treatment outcomes and financial needs in dentistry because of the structural differences between them. The higher the utility value of a health state achieved by a service or treatment strategy, the more deserving it is of being implemented and being allocated resources; establishing the utility of a health state for permanent teeth will help determine the value of dental procedures and utilities. This issue has been discussed in different works of literature [1–5]. Health utility represents individual preference strengths regarding health-related outcomes as a numerical value [6], which also helps in the calculation of quality-adjusted life years (QALY) and acts as a quality adjustment factor that helps in cost-effectiveness and decision analyses [7].

Measuring health state utilities for different health conditions has been more common in the medical field than in dentistry for many years [8–10]. However, in the last decade, research in dentistry has increased; nevertheless, the number of studies that examine professional dentists' preferences remains low. In searching for utility values for different treatment options for restoring the 1st lower molar and upper central region with abscess because of endodontic pathosis, Balevi and Shepperd found higher values with the central option [11].

One of the studies from the field of orthodontics evaluated the quality of life of 108 patients with different grades of malocclusions according to the Index of Orthodontic Treatment Need (IOTN) before and after 12 months of treatment, and the patients with grade 4 showed significant statistical differences in the oral health impact profile (OHIP)-14 [12]. The Early Childhood Oral Health Impact Scale (ECOHIS) is another method used to evaluate oral health-related quality of life in children. A study by Maria Contaldo and her colleagues, which included 87 children, concluded statistical significance between the ECOHIS score and dmft scores when dmft = 0 versus dmft ≥4 [13].

In recent years, patients have had access to health-related information from the Internet, including social media platforms, which facilitate the patient health education process, although the quality of the available data could be better. In two studies by Di Stasio, D., and his colleagues, who investigated the quality of information from YouTube videos regarding mouth sores and oral thrush in children, the information about mouth sores was poor, according to the authors [14]. Also, the information about oral thrush was unsatisfactory regarding quality [15].

Another study including 102 participants with tooth loss concluded that people who had anterior tooth loss showed the lowest utility value (0.16), while the highest value for missing posterior teeth was for the upper and lower second molars (0.48 and 0.47, respectively) [16]. These results show the importance of restoring an anterior tooth according to people's preferences compared to a posterior tooth. Regardless, we also need to consider and compare the preferences of the dentist, who is aware of the consequences of posterior tooth loss, with those of the patients.

The decision to determine the resources of each utility must consider the consumers' perspective on the service's value and willingness to pay for it, as well as the dentists' perspective, as they are the service providers and have more knowledge of the prognosis for different tooth states. Difficulties can arise when these opinions are markedly different between the two groups. Thus, it is essential to establish the difference in utility values between the treated population and clinicians. Especially in this new era of teledentistry and remote communication between patients and dentists, which have been discussed and shown their importance and usefulness in several recent studies [17, 18].

This study aimed to determine if it is possible to locate a measurable value for dental health utilities using the Von Neumann and Morgenstern standard gamble [19], which is commonly used to measure utility value in health [20]. The second aim was to determine how individual tooth utilities may be used to produce a meaningful numerical expression of the health value of compromised dentition by comparing the utility values for a group of professionals and a group of patients.

## 2. Materials and Methods

### 2.1. Sample

The study included two sample groups: 50 volunteer dentists who were working as faculty members at the Faculty of Dentistry at the University of Umm Al-Qura in Makkah, KSA, and 50 volunteer adult patients. Because a variety of patients of different ages and nationalities come to this dental school, only patients who can read and communicate well were included to ensure that they fully understood the questionnaire.

### 2.2. Data

**Figure 1:**
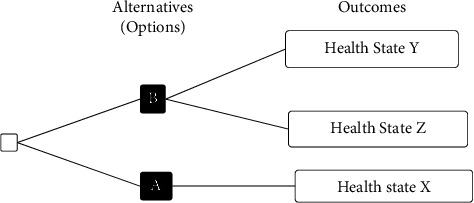
Standard gamble method for measuring health utility value [21].

The two groups were asked to answer a standard gamble questionnaire, which is a classical tool to measure utility value and was constructed to follow the foundations of utility theory [21]. The questionnaire gave participants the chance to choose between different options representing different health states with varying outcomes, one of which includes risk (Figure 1). One of the options (A) would directly lead to a particular health state (*X*) for a certain amount of time, while in the other option (B), the participant was given the probability of having a better health state than in *X*(*Y*) [*p*] and ending in a worse health state than *X*(*Z*) [1 − *p*]. The better health state, *Y*, is usually described as the perfect health condition with a value of 1, and the worse health outcome, *Z*, is described as death with a value of 0 [21].

The participants were asked to answer at what probabilities they would be indifferent between the two alternatives (A) and (B) [22]. When the participant preferred to choose (A) over (B) at a certain probability, this probability was considered to represent the utility value of *X* [23]. This presence of uncertainty and risk when making a health decision is another advantage of using the standard gamble method, as it is similar to making most health-related decisions in life [24].

**Figure 2:**
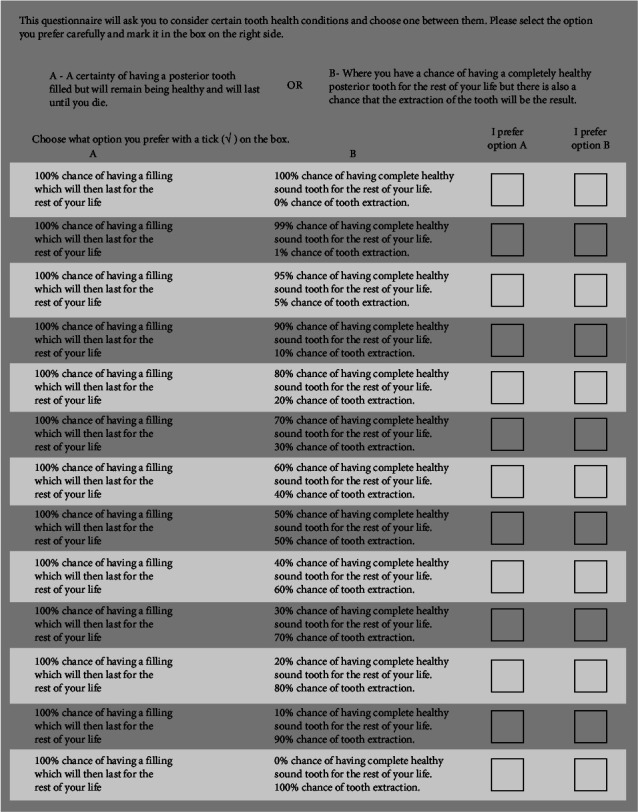
An example of the questionnaire used in this study. The four intermediate tooth health state alternatives were presented as option A one at a time [25].

In this study, we used a questionnaire that was constructed to be used in the dental field from a previous study [25] because we wanted to know participants' preferences regarding specific tooth health outcomes. The best health outcome here was described as normal sound, tooth structure (utility value = 1), while the worst possible outcome, in this case, was tooth extraction (utility value = 0). The participants were able to choose between a specific probability of selecting the perfect health (sound tooth) option that comes with a risk that the worse outcome may happen (extraction) or choosing one of the other hypothetical intermediate tooth state options with a 100% probability that they will end with this option. The four intermediate options were as follows: (1) a carious posterior tooth with pain; (2) a carious posterior tooth without pain; (3) a filled posterior tooth that needs to be restored later; and (4) a filled posterior tooth that does not need more restoration. The four intermediate alternatives replaced option A in the questionnaire (Figure 2) one at a time.

For example, if the participant changes their preference from choosing option A, which is the intermediate health state option with a 100% chance, to choosing the uncertain and risky option B at the point of having a 40% chance of having a healthy sound tooth for the rest of his/her life with a 60% chance of ultimately requiring tooth extraction, then the utility value of option A would be = 0.4 [17].

### 2.3. Statistical Analysis

The recorded data from the interviews and questionnaires were collected, organised, and analysed using a suitable and appropriate statistical test.

## 3. Results

The responses from the 50 patients (35 male, 15 female), aged 15–60 years, were compared with those from 50 volunteer dentists (29 male, 21 female) from various dental specialties.

### 3.1. Comparison between Dentists and Patients

There were significant differences between dentists and patients (Table 1) regarding carious posterior teeth without the pain, filled posterior teeth that need to be restored later, and filled posterior teeth that do not need further restoration, with *p* values of 0.011, 0.026, and 0.008, respectively.

### 3.2. Comparison between Male and Female Participants

Regarding gender differences (Table 2), there was a value gap between male and female participants for the first health state (a carious posterior tooth with pain), with average values of 0.455 and 0.499, respectively. Male participants had the highest value in this comparison for the filled posterior tooth, which does not require more restoration, with an average value of 0.805. However, there were no significant differences between male and female participants for any health states.

### 3.3. Comparison of Dentists and Patients by Gender

The dentist and patient groups were also compared by gender (Table 3). While there was no significant difference between female dentists and patients, there were significant differences between male dentists and patients for a carious posterior tooth with pain and a filled posterior tooth that needs to be restored later, with *p* values of 0.047 and 0.036, respectively.

## 4. Discussion

The study results show measurable values of dentists' preferences regarding permanent dentition in terms of utility. This allowed us to compare it with the same values from people seeking dental treatment (patients). The findings suggest no statistically significant differences between men and women. However, there was a significant difference between dentists and patients regardless of gender in three health states and between male dentists and patients in two health states. Dentists had higher values for all health states.

One of the findings was the possibility of using a utility measurement method, such as the standard gamble, among dentists and dental patients in this subject area. These utility values consider parameters for determining quality-adjusted life years (QALYs); in the case of the dental practice, it is more appropriate to describe them as quality-adjusted tooth years (QATYs), as mentioned by Fyffe and Kay [25]. The utility values are described as QALY-weight, which should be determined at the beginning to calculate QALYs. To explain the role of utilities in this system, we assumed that there were two treatment options for a specific tooth (as in our study survey). Both treatments extend the expected life years of the tooth by 5 years; treatment A results in passing the 5 years with a health state with an assigned value of 0.7 (usually assigned value of 1), while treatment B results in passing the 5 years with a surviving tooth but with a health state (e.g., carious posterior tooth with pain) with an assigned value of 0.5 (the answer from the questionnaire). In this case, treatment A will gain 5 years in QATYs (5 × 1), while treatment B will gain 3.5 years (5 × 0.7 = 3.5 years) [6].

This means that a higher utility value from a person regarding a specific tooth health state will show greater tooth longevity based on the QATYs.

The role of utilities in QALYs eventually leads to cost-utility analysis, representing a method of economical medical (dental) assessment. It allows health economists to compare different types of medical treatment procedures or alternatives that usually do not have common criteria for comparison (e.g., endodontics and restorative dental treatments) by valuing each of them with a standard measuring unit, the utility value. This helps with resource allocation in dental services, including clinical and social interventions. QALYs are not the only method that uses utilities for economic health evaluation; it includes others such as health year equivalents (HYEs), which have been tested and failed in dentistry [26].

The finding that the QATYs-weight (utility) given by dentists was higher in this study was consistent with the findings of Fyffe & Kay, who compared dentists and the general public [19]. However, this study showed a less significant difference between dentist and dental patient values. In this case, we compared patients who already had dental issues and had/have dental needs that made them seek dental care. We assumed that higher utility values from patients in our study might indicate their personal experience of experiencing dental diseases or treatment procedures (e.g., tooth extraction), as concluded in a previous study [27], which does not necessarily exist in the general public.

Differences and similarities between patient and dentist preferences could also provide insights into the level of patient education regarding dental health and dental treatment options. Because the standard gamble theory depends on participant risk behaviour as a significant factor for the interpretation of the results [28], it is difficult to neglect the effects of patient dmfts scores and oral health histories on the result, and this effect may extend to include patient age and socioeconomic status.

By paying attention to all these aspects, decision-makers and dentists, including dental college faculty members, could use the values of patient preferences to improve the evidence-based decision-making process that includes, for example, community dental health policy development or undergraduate dental students' education, extending to other dental economic implications such as measuring patients' payment for dental treatment in private dental services [29].

This study was limited to measuring the preference of adult patients for health states related to their permanent dentition without determining the effects of individual dental histories or demographic factors. The study sample was also limited to faculty members and patients at UQUDENT clinics, and there was a relatively small number of participants, which may limit the generalizability of the findings. Difficulties in communication and in describing the study's aim and the questionnaire were obstacles for some participants.

Dental economics remains a new field of research. Providing more information to dentists, even during the undergraduate stay, could help in conducting more studies that may use a larger sample size or investigate the preferences of specific types of patients (e.g., handicapped, elderly, or other medical staff) in the dental field, which will lead to the effective allocation of dental resources at the clinical, social, and educational levels.

## 5. Conclusion

The oral health-related quality of life and its relation to economic dentistry is essential aspects of our modern practice. This study conducted this concept in a population where this type of study is still new and unfamiliar to many patients and dentists. The findings show a statistical difference in utility value between dentists and patients. However, more research is needed in this area.

## Figures and Tables

**Table 1:** Comparison between dentists and patients regarding health status.

Health state	Dentists (50) mean ± SD	Patients (50) mean ± SD	*p*
Carious posterior tooth with pain	0.516 ± 0.304	0.425 ± 0.340	0.162
Carious posterior tooth without pain	0.724 ± 0.256	0.584 ± 0.283	0.011^*∗*^
Filled posterior tooth, which needs to be restored later	0.732 ± 0.209	0.626 ± 0.259	0.026^*∗*^
Filled posterior tooth, which does not need further restoration	0.852 ± 0.148	0.752 ± 0.215	0.008^*∗*^

*p* values were calculated using the unpaired Student's *t*-test, ^*∗*^Significant *p* value.

**Table 2:** Comparison between male and female participants regarding health status.

Health state	Male participants (64) mean ± SD	Female participants (36) mean ± SD	*p*
Carious posterior tooth with pain	0.455 ± 0.353	0.499 ± 0.270	0.525
Carious posterior tooth without pain	0.649 ± 0.284	0.663 ± 0.270	0.800
Filled posterior tooth, which needs to be more restored later	0.683 ± 0.245	0.672 ± 0.233	0826
Filled posterior tooth, which does not need more restoration	0.805 ± 0.196	0.796 ± 0.182	0.826

*p* values were calculated using the unpaired Student's *t*-test.

**Table 3:** Comparison of dentists and patients by gender.

Health state	Dentists		Patients		*p*
	Males (29) mean ± SD	Females (21) mean ± SD	Males (35) mean ± SD	Females (15) mean ± SD	
Carious posterior tooth with pain	0.566 ± 0.326^A^	0.447 ± 0.263	0.363 ± 0.351^A^	0.570 ± 0.272	0.047^*∗*^
Carious posterior tooth without pain	0.747 ± 0.243	0.692 ± 0.277	0.567 ± 0.293	0.623 ± 0.265	0.064
Filled posterior tooth, which needs to be more restored later	0.778 ± 0.167^B^	0.669 ± 0.246	0.605 ± 0.273^B^	0.677 ± 0.223	0.036^*∗*^
Filled posterior tooth, which does not need more restoration	0.866 ± 0.131	0.832 ± 0.171	0.755 ± 0.227	0.747 ± 0.191	0.062

*p* value was calculated using a one-way analysis of variance; A, B: similar letters indicate a significant difference between corresponding columns calculated using the post hoc *t*-test, ^*∗*^Significant *p* value.

## Data Availability

The data used to support the findings of this study are available from the corresponding author upon reasonable request.
